# Effects of Zearalenone on IL-2, IL-6, and IFN-**γ** mRNA Levels in the Splenic Lymphocytes of Chickens

**DOI:** 10.1100/2012/567327

**Published:** 2012-05-02

**Authors:** Y. C. Wang, J. L. Deng, S. W. Xu, X. Peng, Z. C. Zuo, H. M. Cui, Y. Wang, Z. H. Ren

**Affiliations:** ^1^College of Veterinary Medicine, Sichuan Agricultural University, Yaan, Sichuan 625014, China; ^2^Department of Veterinary Medicine, Northeast Agricultural University, Harbin 150030, China; ^3^Sichuan Key Laboratory of Animal Disease and Human Health, Yaan, Sichuan 625014, China; ^4^Sichuan Key Laboratory of Environmental Hazards Disease, Yaan, Sichuan 625014, China

## Abstract

Zearalenone (ZEN) is an estrogenic mycotoxin produced by several *Fusarium* species, which can contaminate food and feed. These compounds elicit a wide spectrum of toxic effects, including the capacity to alter normal immune function. In this study, the in vitro effects of the treatment of ConA-stimulated splenic lymphocytes with ZEN (0–25 *μ*g/mL) were examined. ZEN modulates the expression of IL-2, IL-6, and IFN-*γ*. The IL-2 levels were up to fourfold higher (*P* < 0.05) compared with the levels in the control at toxin concentrations of 25 *μ*g/mL after 48 h of treatment. The IL-6 levels were critically suppressed at this concentration; these changes were very statistically significant (*P* < 0.05). At lower ZEN concentrations (0.1, 0.4 and 1.6 *μ*g/mL), the IFN-*γ* levels changed slightly; however at 6.25 and 25 *μ*g/mL, the IFN-*γ* results reached statistical significance compared with the control levels (*P* < 0.05). These data suggest that ZEN has potent effects on the expression of chicken splenic lymphocytes cytokines at the mRNA level.

## 1. Introduction

Zearalenone (ZEN) is a mycotoxin produced by several field fungi, including *Fusarium graminearum* (Gibberella zeae), *F. culmorum*, *F. cerealis*, *F. equiseti*, and *F. semitectum* [1, 2]. It exists widely in many cereal crops such as corn, barley, wheat, oats, sorghum, and sesame seeds, as well as in hay and corn silage. These are all ingredients in many food products for human or animal nutrition [3–5].

ZEN is a macrocyclic lactone with a high binding affinity for estrogen receptors. It is biologically potent, but it is hardly toxic. Rather, it has an estrogenic effect that causes alterations in the reproductive tract of laboratory animals (mice, rats, and guinea pigs) and farm animals [6, 7]. The mechanism of the estrogenic effects of ZEN appears to be mediated via binding of this mycotoxin or its metabolites to the cytoplasmic estrogen receptor [8–10]. This increases cell proliferation [11], resulting in uterine hyperplasia as well as cervical and vaginal metaplasia [12, 13].

The immune system is a potential target for estrogenic endocrine disruptors because various cells of the immune system have estrogen receptors [14]. However, only few studies have been carried out regarding the effects of ZEN on spleen immunity in chickens, specifically on lymphocytes. In the present study, we investigated the in vitro effects of ZEN at different concentrations on IL-2, IL-6, and IFN-*γ* mRNA expression levels in poultry spleen lymphocytes.

## 2. Materials and Methods

### 2.1. Reagents

All chemicals were of the highest grade of purity available. Fetal bovine serum (FBS) was purchased from Sijiqing Biological Engineering Material (Hangzhou, China). Zearalenone, RPMI 1640 medium, and Histopaque 1077 were purchased from Sigma-Aldrich, USA. Trizol reagent was purchased from Invitrogen Biotechnology Co. Ltd. (Shanghai, China). SYBR PremixScript RT-PCR Kit II was purchased from Takara, Shiga, Japan.

### 2.2. Cell Culture

All chickens used in this experiment were approved by the Institutional Animal Care and Use Committee of Northeast Agricultural University. The spleens of two-month-old *Isa Brown *chicken were teased through a 200-mesh cell strainer into a Petri dish containing phosphate-buffered saline (PBS). The cell suspension was overlaid onto Histopaque 1077 and was centrifuged at 400 ×g for 15 min at room temperature. The lymphocytes at the interface were collected, washed twice with PBS at 250 g for 5 min at room temperature, and suspended in RPMI-1640 medium (without phenol red, a weak estrogen mimic) supplemented with 10% fetal calf serum, 100 U/mL penicillin, 100 U/mL streptomycin. More than 95% of cells were viable based on the trypan blue dye exclusion. The spleen cells were cultured in 6-well tissue culture plates (6 × 10^6^ cell/mL) in triplicate and stimulated with concanavalin A (ConA, 12.5 *μ*g/mL, to induce the proliferation of T cells) at 0, 0.1, 0.4, 1.6, 6.25, and 25 *μ*g/mL ZEN at 41.5°C in a humidified 5% CO_2_ environment for 48 h [15]. The mycotoxin concentrations were selected based on preliminary dose-response experiments (data not shown). After 48 h of incubation, cells were collected for RNA isolation, then frozen at −80°C.

### 2.3. Quantification of IL-2, IL-6, and IFN-*γ* mRNA

Total RNA was isolated from cells using Trizol reagent according to the instructions of the manufacturer. The RNA concentrations were determined using the GeneQuant 1300.

The 40 *μ*L reverse transcription reaction mixture consisted of the following components: 10 *μ*g of total RNA, 1 *μ*L of Moloney murine leukemia virus reverse transcriptase, 1 *μ*L of RNase inhibitor, 4 *μ*L of deoxynucleoside triphosphate, 2 *μ*L of Oligo dT, 4 *μ*L of dithiothreitol, and 8 mL of 5x reverse transcriptase buffer. The reverse transcription was performed according to the instructions of the manufacturer (Invitrogen). The reverse transcription products (cDNA) were stored at −20°C for PCR.

To design primers, we used the chicken IL-2, IL-6, and IFN-*γ* mRNA GenBank sequence with accession numbers of NM204153.1, NM204628.1, and DQ470471.1, respectively. Chicken *β*-actin (GenBank accession number L08165.1), a housekeeping gene, was used as the internal reference. The primers (Table 1) were designed using the Prime 5 Software (Molecular Biology Insights, Cascade, CO) and were synthesized by Invitrogen Biotechnology Co. Ltd., Shanghai, China.

Real-time PCR was performed to detect the expression of IL-2, IL-6, and IFN-*γ* genes in different cDNA sample using SYBR Premix Ex Taq (Takara, Shiga, Japan). Each sample was assayed in three replicates. The reaction mixtures were incubated in an ABI PRISM 7500 real-time PCR system (Applied Biosystems, Foster City, CA). The program included 1 cycle at 95°C for 30 s, 40 cycles at 95°C for 5 s, and 60°C for 34 s. The dissociation curves were analyzed using Dissociation curve 1.0 Software (Applied Biosystems) for each PCR reaction to detect and eliminate the possible primer dimer and nonspecific amplification. Results (fold changes) are expressed using the Pfaffl method [16, 17] with the following formula:

(1) $$\text{Ratio}=\frac{{\left({\text{E}}_{\text{target}}\right)}^{\Delta \text{CT},\;\;\text{target}\;\;(\text{calibrator-test})}}{{\left({\text{E}}_{\text{ref}}\right)}^{\Delta \text{CT},\;\;\text{ref}\;\;(\text{calibrator-test})}}$$

where ΔCT,   target  (calibrator-test) = (CT_target_)_control  group_ − (CT_target_)_treatment  group_,   ΔCT, ref  (calibrator-test) = (CT_ref_)_control  group_ − (CT_ref_)_treatment  group_, E_target_ is the real-time PCR efficiency of target gene transcript, and E_ref_ is the real-time PCR efficiency of a reference gene transcript.

### 2.4. Statistical Analysis

Statistical analysis of all data was performed using SAS procedures (SAS Institute Inc, NC); differences in mean RT-PCR results were compared by one-way ANOVA. All values are expressed as mean ± SD, and *P* < 0.05 were considered as significant difference.

## 3. Results

**Figure 1:**
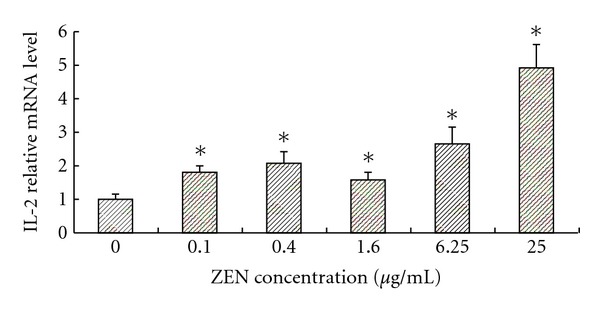
The effect of ZEN on the IL-2 relative mRNA level in ConA-stimulated lymphocytes at toxin concentration of 0, 0.1, 0.4, 1.6, 6.25, and 25 *μ*g/mL at 48 h. The reporter activity in response to ConA alone is expressed as 100%. Values are the means ± SD from three independent experiments. Statistical analysis was performed using one-way ANOVA followed by Dunnett's post hoc test (_  _*P < 0.05 versus ConA treated control).

**Figure 2:**
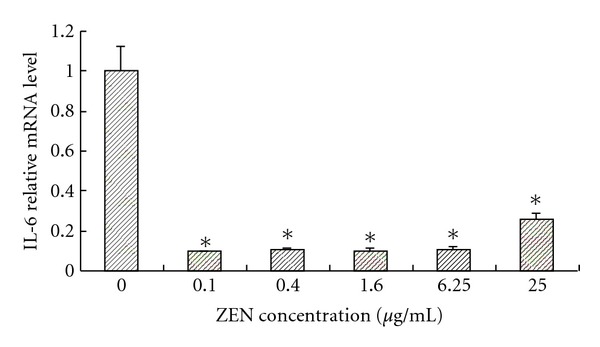
The effect of ZEN on the IL-6 relative mRNA level in ConA-stimulated lymphocytes at toxin concentrations of 0, 0.1, 0.4, 1.6, 6.25, and 25 *μ*g/mL at 48 h. The reporter activity in response to ConA alone is expressed as 100%. Values are the means ± SD from three independent experiments. Statistical analysis was performed using one-way ANOVA followed by Dunnett's post hoc test (_  _*P < 0.05 versus ConA treated control).

To compare the effects of ZEN on cytokine expression in residual living cells among individuals, the results were calculated as mean fold changes in cytokine levels above the control (treated with only ConA) cultures at the same time points. Treatment of the ConA-stimulated cells with ZEN at concentrations ranging from 0 to 25 *μ*g/mL consistently increased the IL-2 levels above those in the ConA-only stimulated cells (Figure 1). These results were statistically significant (*P* < 0.05) with toxin concentrations of 0.1, 0.4, 1.6, 6.25, and 25 *μ*g/mL at 48 h (Figure 1). In contrast, the ZEN treatment moderately inhibited IL-6 levels in these cells; these changes were very statistically significant with toxin concentration in all treated groups (*P* < 0.05). The IL-6 levels were critically suppressed (Figure 2).

**Figure 3:**
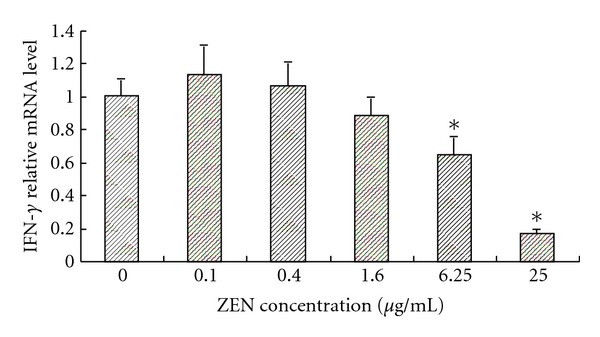
The effect of ZEN on the IFN-*γ* relative mRNA level in ConA-stimulated lymphocytes at toxin concentrations of 0, 0.1, 0.4, 1.6, 6.25, and 25 *μ*g/mL at 48 h. The reporter activity in response to ConA alone is expressed as 100%. Values are the means ± SD from three independent experiments. Statistical analysis was performed using one-way ANOVA followed by Dunnett's post hoc test (_  _*P < 0.05 versus ConA treated control).

To understand further the specific effects of ZEN on cytokine profiles, a characteristic TH 1 cytokine, IFN-*γ*, was measured in addition to IL-2 and TH 2, IL-6. A lower dose of ZEN (0.1, 0.4, and 1.6 *μ*g/mL) was selected to eliminate the possibility of cytotoxic effects, which contributes to modulation; however, the two highest doses (6.25 and 25 *μ*g/mL) reached statistical significance compared with the control (*P* < 0.05). The IFN-*γ* levels were slightly suppressed by the ZEN treatment (Figure 3).

## 4. Discussion

There is major concern over the potential for mycotoxins to influence poultry negatively. In the present study, the use of cell cultures offers several advantages over other methods, particularly in terms of the quantification of toxic effects and for defining the immune organ specificity related to a preferential action of a particular cell type. We addressed the issue by evaluating the effects of ZEN on the immune function through in vitro assessment of its activity on the splenic lymphocytes of chickens. The splenic lymphocytes of chickens have been a very useful model for different immunologic studies. ConA is a polyclonal T-cell mitogen, and we determined the effects of ZEN on ConA-stimulated lymphocytes on certain T-helper cell (TH), cytokine profiles (IL-2, IL-6, and IFN-*γ*) were assessed. 

To date, there have been no studies regarding the effects of ZEN on the cytokine profiles of chicken splenic lymphocyte. The addition of ZEN in vitro to ConA-stimulated lymphocytes caused a fivefold increase in the expression of IL-2 cytokines at the mRNA level. As the chemical structure of ZEN enables its binding to the estrogenic receptors, our data are not in line with previous results, which indicate that 17-beta-estradiol reduced IL-2 gene transcription in the Jurkat T-cell line [18]. Considering that T-lymphocyte proliferation is essentially mediated by IL-2, the observed inhibition could be considered a primary mechanism of ZEN-induced immunosuppression also acting in vivo. Accordingly, inhibition of the mRNA expression of IL-2 cytokines by *α*-ZEN remain unclear assuming an estrogen-like activity [18]. Our results on IL-2 are not broadly in agreement with studies on cytokine protein expression in humans [19]. However, several alterations in immunologic parameters have been associated with ZEN concentrations in humans [20]and mice [21] including in vitro inhibition of mitogen-stimulated lymphocyte proliferation, increase of IL-2 and IL-5, and induction of immunosuppressive effects [22, 23]. Alterations in immunologic parameters such as the inhibition of mitogenstimulated lymphocyte proliferation and the increase in IL-2 production were found at high ZEA concentrations in vitro [22]. In addition, populations that are continuously exposed to ZEN may chronically produce IL-2; therefore, IL-6 is decreased. There are only few reports regarding the effects of ZEN on IL-6. Aside from ZEN, *Fusarium* can also produce other mycotoxins such as deoxynivalenol (DON), fumonisin B1 (FB1), and T-2 toxin. The effect of these mycotoxins on IL-6 has been studied. A study [24] quantified IL-6 in the spleen, ileum, and mesenteric lymph nodes of 24 pigs which received either control feeds or feeds naturally contaminated with 2.2–2.5 mg/kg of DON feed for 9 weeks using RT-PCR. FB_1_ inhibited the LPS-induced expression of IL-6 in human dendritic cells (DCs) [25], whereas the modulation of FB_1_ toxicity in the brain of female BALB/c mice; FB_1_ augments the LPS-induced IL-6 expression in the brain [26]. The effects of in vivo exposure to T-2 toxin on the alteration of IL-6 in lipopolysaccharide-stimulated peritoneal macrophages [27]; IL-6 mRNA from activated peritoneal macrophages showed no significant differences between the control and the treatment groups. The effects of different *Fusarium *toxins on IL-6 varied because of differences in toxicities and animal models. 

IFN-*γ* is a master cytokine that affects the functioning of all cells of the immune system and plays a major role in host defense against intracellular infections [28]. This cytokine has also been implicated in autoimmune and inflammatory diseases [29]. Importantly, the IFN-*γ* levels in ConA-activated splenic lymphocytes that were exposed to zearalenone decreased after 48 h of culture, consistent with previous findings. Studies revealed that zearalenone causes a decrease in IFN-*γ* in geriatric mice [30], and a slight reduction in both the mitotic index and the cell survival of bovine lymphocytes [31]. 

To our knowledge, this is the first study that investigates the effects of ZEN on the cellular immune response of chickens. Although the exact mechanism of action of these toxins is still unknown, the results of the current study suggest that ZEN may have divergent effects on chicken lymphocyte cell viability, and production of IL-2, IL-6 and IFN-*γ*. Further studies are needed to elucidate the specific mechanisms by which ZEN affects immune functions.

## Figures and Tables

**Table 1:** Real-time PCR primer sequences and products.

Gene	Primer	Sequences (5′→3′)	Product size (bp)
*β*-actin	*β*-Actin Forward	CACCACAGCCGAGAGAGAAAT	135
	*β*-Actin Reverse	TGACCATCAGGGAGTTCATAGC	
IL-2	IL-2 Forward	GCTAATGACTACAGCTTATGGAGCA	138
	IL-2 Reverse	TGGGTCTCAGTTGGTGTGTAGAG	
IL-6	IL-6 Forward	AAATCCCTCCTCGCCAATCT	106
	IL-6 Reverse	CCCTCACGGTCTTCTCCATAAA	
IFN-*γ*	IFN-*γ* Forward	AAGTCATAGCGGCACATCAAAC	132
	IFN-*γ* Reverse	CTGGAATCTCATGTCGTTCATCG	
